# Cooktop Sensing Based on a YOLO Object Detection Algorithm

**DOI:** 10.3390/s23052780

**Published:** 2023-03-03

**Authors:** Iker Azurmendi, Ekaitz Zulueta, Jose Manuel Lopez-Guede, Jon Azkarate, Manuel González

**Affiliations:** 1 Department of Systems and Automatic Control, Faculty of Engineering of Vitoria-Gasteiz, University of the Basque Country (UPV/EHU), Nieves Cano, 01006 Vitoria-Gasteiz, Spain; 2 CS Centro Stirling S. Coop., Avda. Álava 3, 20550 Aretxabaleta, Spain

**Keywords:** deep learning, artificial vision, object detection, YOLO, YOLOv5, YOLOv6, YOLOv7, cooking automation, smart kitchen, image sensorization

## Abstract

Deep Learning (DL) has provided a significant breakthrough in many areas of research and industry. The development of Convolutional Neural Networks (CNNs) has enabled the improvement of computer vision-based techniques, making the information gathered from cameras more useful. For this reason, recently, studies have been carried out on the use of image-based DL in some areas of people’s daily life. In this paper, an object detection-based algorithm is proposed to modify and improve the user experience in relation to the use of cooking appliances. The algorithm can sense common kitchen objects and identify interesting situations for users. Some of these situations are the detection of utensils on lit hobs, recognition of boiling, smoking and oil in kitchenware, and determination of good cookware size adjustment, among others. In addition, the authors have achieved sensor fusion by using a cooker hob with Bluetooth connectivity, so it is possible to automatically interact with it via an external device such as a computer or a mobile phone. Our main contribution focuses on supporting people when they are cooking, controlling heaters, or alerting them with different types of alarms. To the best of our knowledge, this is the first time a YOLO algorithm has been used to control the cooktop by means of visual sensorization. Moreover, this research paper provides a comparison of the detection performance among different YOLO networks. Additionally, a dataset of more than 7500 images has been generated and multiple data augmentation techniques have been compared. The results show that YOLOv5s can successfully detect common kitchen objects with high accuracy and fast speed, and it can be employed for realistic cooking environment applications. Finally, multiple examples of the identification of interesting situations and how we act on the cooktop are presented.

## 1. Introduction

In recent years, Artificial Intelligence (AI) has experienced monumental growth, becoming a powerful tool that allows machines to think and act like humans. One of its most studied areas is computer vision, which aims to capture images of the real world, process them, and generate information for analysis. In addition, although advances in computer vision have been built and refined over time, nowadays, Deep Learning (DL) techniques are the most widely used for computer vision because they provide a spectacular performance improvement compared to traditional image processing algorithms. Examples of the use of computer vision include image and video recognition, image analysis and classification, recommendation systems, and natural language processing [1].

There are different computer vision algorithms based on DL, such as image classification, object detection and image segmentation. On the one hand, image classification divides images into various categories and groups. On the other hand, object detection refers to the identification and localization of multiple objects within an image. Finally, image segmentation is a per-pixel classification process that assigns a category to each pixel of the analyzed image. The choice of an algorithm will depend on the level of analysis of the image, also considering the execution times. In this work, an object detection algorithm will be used since the analysis of images with bounding boxes is sufficient for the task at hand.

Object detection and recognition using Neural Networks (NN) and DL is a hot topic in computer vision, and it is a very useful capability for automation, robotics, and intelligent applications [2]. The problem definition of object detection is to determine where objects are in a given image (object localization) and which category belongs to each object (object classification) [3].

These types of algorithms are being used in many fields, such as agronomy [4,5], augmented reality [6,7], autonomous navigation [8,9] and Unmanned Aerial Vehicles (UAVs) [10,11]. However, it is difficult to find any research that uses this technology to detect and recognize common household objects in realistic environments, even though it is one of the key factors for service robotics. Therefore, this paper focuses on one of these areas, the kitchen, and more precisely, the cooking environment, with the purpose of helping users.

The state of the art of computer vision in cooking environments shows that this technology is used for tasks such as cooking state recognition [12], collaborative cooking [13], and assistive cooking using augmented reality [6]. However, this study will show that by combining artificial vision with deep learning, further improvements in cooking automation, safety and energy efficiency can be achieved.

Nowadays, cooking solutions for the domestic and professional markets lack elements that allow for feedback of the situation in the kitchen. The traditional approach has consisted of integrating basic sensors, generally temperature sensors, to identify the elements present in the kitchen (type of kitchenware and contents, such as water or oil) and the evolution of the cooking process (temperature of the contents of the container). The objective was to provide users with valuable information that could improve the use and experience of kitchens, as well as achieve a more efficient and safer operation. This type of approach has some disadvantages: the solutions are particular to each firing technology (glass-ceramic or induction), and therefore, the type of information that can be obtained is limited.

In this way, the objective of this work is to study and develop a sensor fusion technology that, based on a vision algorithm (in this case, an object detection algorithm), will help to modify and improve the user experience in relation to the use of kitchens. Moreover, the object detection algorithm will allow for real-time analysis, associating the received image with interesting situations for users, and acting quickly and directly on the cooktop. Some of these interesting situations could be the following:

- Presence or non-presence of utensils on lit hobs

This is useful, for example, in situations where a cooker is accidentally left on or forgotten. The system could emit an alarm signal and prevent a risky situation or simply avoid a situation of wastage. If the cooker has connectivity, it would even be possible to switch it off. This is of particular interest for groups with special needs (e.g., Alzheimer’s) or for the impact on energy savings (e.g., the beeping of the fridge when the door is left open).

- Boiling/smoking

It would be helpful to identify a smoking/boiling situation in the cooking process so that, for instance, the fire power could be lowered to make cooking more efficient, or the user could be warned that something is burning on the fire. In addition, different cooking states/situations could be detected by trying to automate the whole cooking process.

- Presence of user manipulating the cookware

This feature is likely to improve the safety of the kitchen. In the case of a domestic kitchen, it could detect children accidentally touching the cooker, emitting an audible signal or even sending a message to a mobile phone.

- Adjusting the size of the pan

Inappropriate situations with considerable differences in size between the cookware used and the cooking source can be detected and notified to optimize their use.

For the implementation of this work, a camera will be installed in the kitchen to recognize its situation of use, being able to give feedback to the user and the system. To achieve this goal and to robustly identify objects of different classes, the system needs to be trained with many images of multiple objects in different situations.

In addition, the family of pre-trained object detection architectures of YOLOv5 [14] will be used. This version of YOLO offers different heavyweight models that vary in terms of the number of model parameters, and therefore, in their accuracy and inference time. Nevertheless, other object detection algorithms will also be studied and compared to determine which of them gives better results, such as the new YOLOv6 [15] and YOLOv7 [16] algorithms.

The following is an overview of the research contributions:

- A DL algorithm using a visual sensor is presented for smart control of the cooktop.
- An algorithm that can automatically send commands to the cooker has been developed and implemented in a real cooking environment.
- A robust dataset consisting of more than 7500 images has been developed.
- Different data augmentation techniques have been applied and the results have been compared.
- Different types of models have been trained on the dataset and their comparative has been presented. The indexes of average precision (AP), mean average precision (*mAP*), model size, frames per second (FPS), and training time are investigated.
- The results show that an object detection algorithm can be used to improve the user experience in a cooking environment.

The rest of the paper is organized as follows. Section 2 presents the DL and YOLO object detection algorithms. Section 3 shows the used datasets and discusses the data augmentation techniques applied to improve the results. The training results, comparing different data augmentation techniques and object detection models, are discussed in Section 4. Moreover, some examples of the considered interesting situations are illustrated. The results are discussed in Section 5. We conclude the paper in Section 6.

## 2. Materials and Methods

### 2.1. Deep Learning

Machine Learning (ML) and AI are becoming the dominant problem-solving techniques in many areas of industry and research, particularly due to the recent success of DL. The world is growing exponentially, and the size of the data created, captured, copied, and consumed around the world is too. These data are becoming increasingly meaningful and contextually relevant, opening up new opportunities for such techniques [17].

According to Mitchell (1997) [18], ML is the science that “addresses the question of how to build computer programs that automatically improve with experience”. Therefore, both AI and ML try to build intelligent computer programs, and DL, as a case of ML, is not an exception [15].

DL [19,20] is a type of ML that is essentially an NN with three or more layers. These neural networks attempt to simulate the behavior of the human brain by allowing it to “learn” from large amounts of data. In recent years, DL has made significant advances in domains such as image and video recognition or classification [21], autonomous systems and robotics [22], text analysis and natural language processing [23] and medicine [24], among many others [25].

**Figure 1:**
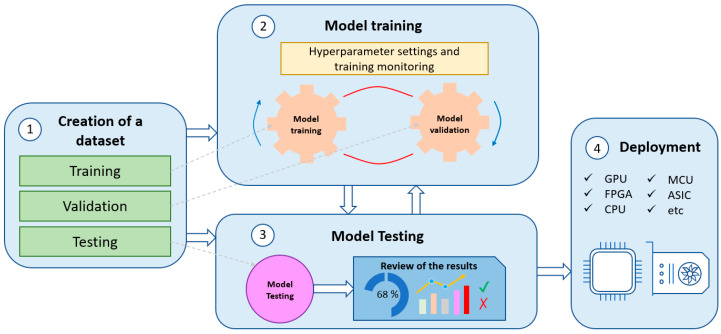
Deep Learning workflow.

DL consists of four phases: (a) the creation of a dataset (if there is no open-access dataset available), which allows for characterizing the problem to be solved, and therefore, obtaining accurate results; (b) training, where the input data are used to calculate the model parameters; (c) the evaluation phase, where the trained model gives a value to different input samples and the algorithm is evaluated; and (d) the deployment and execution of the model for a specific application, e.g., on a microcontroller or a Graphics Processing Unit (GPU). Figure 1 shows the described workflow for DL.

### 2.2. Object Detection

As mentioned in the Introduction, due to recent advances in AI techniques, computer vision and DL have gained a lot of attention. Applications that were considered extremely difficult several years ago have become much easier to create, offering extremely good accuracy and runtime results. One of these applications is object detection.

Object detection, one of the fundamental challenges of computer vision, seeks to locate instances of objects among a set of predefined categories in natural images [26,27]. Generic object detection, also called generic object category detection, category detection or object class detection, is the detection of object instances of a specific category in images, videos, or even real-time images. The key element in this type of model is the bounding box associated with each of the categories identified in the input images.

DL-based object detection models use neural network architectures such as YOLO (You Only Look Once), SSD (Single Shot Multibox Detector), RetinaNet, RCNN (Region-based CNN), etc., for object feature detection to perform characterization to a given category. These object detection models are generally characterized into two groups: single-stage detectors such as YOLO and SSD or two-stage detectors such as RCNN.

### 2.3. YOLO Architecture

YOLO is one of the most popular object detection algorithms and model architectures. YOLO is a type of 1-stage detector in which the regional proposal and classification are performed simultaneously, i.e., it proposes the use of an end-to-end neural network that performs bounding box predictions and class probabilities at the same time. In general, the deeper the CNN, the better the performance. However, as the network gets deeper, the number of parameters to be learned increases, leading to longer training times. On the other hand, YOLO works by dividing the images into a grid system, where each grid is loaded to detect objects within it. Moreover, non-maximum suppression is performed, ensuring that the object detection algorithm only identifies each object once. Consequently, YOLO is one of the most famous object detection algorithms due to its speed and accuracy.

The original YOLO model was the first object detection network to combine the problem of drawing bounding boxes and identifying class labels in one end-to-end differentiable network. In addition, due to its flexible research framework written in low-level languages, different variations of the algorithm have been produced: YOLOv1, YOLOv2, YOLOv3, YOLOv4, YOLOv5, YOLOv6, YOLOv7, YOLOX, YOLOR and YOLOP.

Version 5 of the YOLOv5 algorithm will be used for this work due to its good results and its simplicity of implementation. Furthermore, it allows us to choose different models from the same version depending on the characteristics of the application being developed (accuracy and inference time).

YOLOv5 is a family of object detection architectures and models pretrained on the COCO dataset. The YOLOv5 is a state-of-the-art, single-stage, real-time object detector based on the YOLOv1, YOLOv2, YOLOv3, and YOLOv4 models [28]. The continuous developments in the model architecture have resulted in top performances on two of the biggest official datasets: the Pascal Visual Object Classes (VOC) [29] and Microsoft Common Objects in Context (COCO) [30].

### 2.4. Automatic Cooktop Control Algorithm

The flowchart of the cooktop control algorithm starts with the training, validation, and testing of a DL model. The training and validation of the object detection algorithm is done with one of the datasets discussed in Section 3. The testing of the model will be performed on a real functional mock-up in which the generalizability of the algorithm to new objects is determined. The resulting DL model will be used as the basis for the real-time execution algorithm. This algorithm will perform the object detection task and the mentioned interesting situations will be identified. Depending on these situations, automatic control of the cooktop will be carried out.

However, before running the real-time algorithm that detects those situations and acts on the cooker hob, an initialization is necessary. This initialization consists of switching on the cooker and connecting it to the program. On the one hand, the determination of whether the cooker is on or off is carried out by a convolutional neural network. On the other hand, in the case of detecting that the cooker is on, the computer or device will be connected to the cooker via a Bluetooth Low Energy (BLE) module. Once the computer is connected to the cooker, it will start running the real-time algorithm.

The following algorithm works as follows: an image is captured, processed and the available objects are detected. From the available objects, situations of interest are identified. Some of these situations will involve acting on the cooker, so, if necessary, appropriate commands will be generated and sent to it.

**Figure 2:**
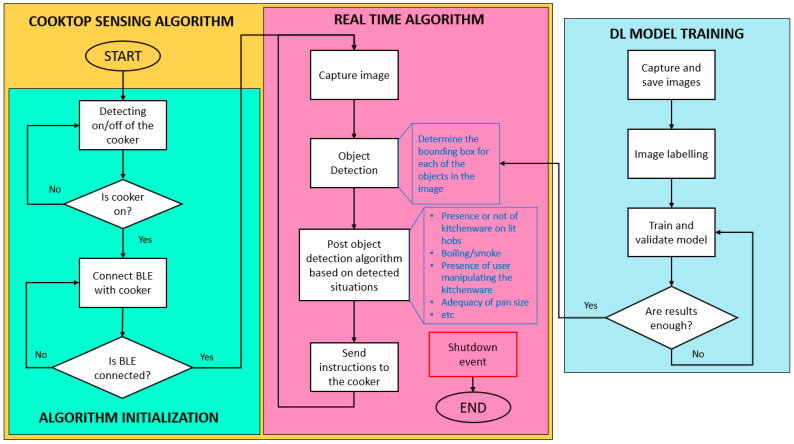
Automatic cooktop control algorithm workflow.

The workflow of the complete automatic cooktop control algorithm is shown in Figure 2.

## 3. Datasets

### 3.1. Importance of the Training Data

In DL, as in ML, one of the most important considerations is the type of data you give to the model. If there is more data, there is a better chance that an ML algorithm will understand it and give accurate predictions to previously unseen data.

As referenced by Li Liu et al. [26], datasets have played a critical role in the history of deep learning research, not only as a common ground for measuring and comparing the performance of different competing algorithms, but also for pushing the field toward increasingly complex and challenging problems. For example, access to many images on the internet allows the construction of large datasets for different fields, enabling unprecedented performance for computer vision algorithms.

There can be many forms of data that can be used for ML. However, some of the principal types that are given to these algorithms for making predictions are categorical data, numerical data, time series data and text data.

The first step in the process of ML, and therefore, of DL, is data preparation. Xin He et al. [31] present a workflow for data preparation, which can be summarized in three steps: data collection, data cleaning and data augmentation. First, data collection is a necessary step to build a new dataset or extend an existing one. The data cleaning process is used to filter out noisy data to avoid compromising the training of the model. Finally, data augmentation plays an important role in improving the robustness and performance of the model. After going through the data preparation process, you are ready to train your DL model.

### 3.2. Data Description

**Figure 3:**
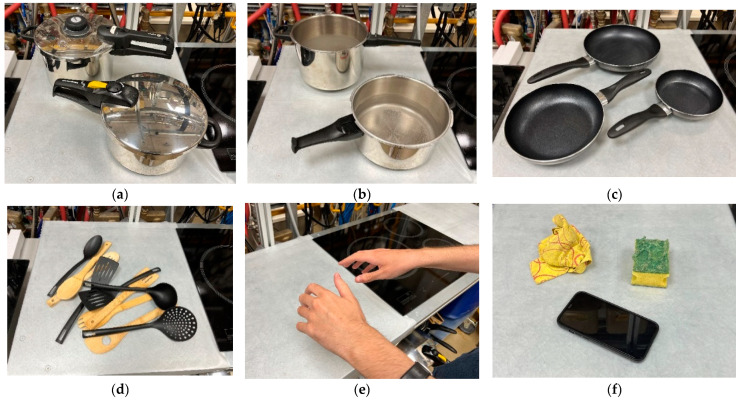
Object detection dataset classes: (**a**) Close pots; (**b**) Open pots; (**c**) Pans; (**d**) Kitchenware; (**e**) User; and (**f**) Others.

The training data used in this work are images of everyday objects in a real cooking environment. The total number of images corresponding to the main dataset is more than 7500, and all are in JPG format. The images have been manually labelled and provide relevant information about the application environment and the situations to identify. In total, eight objects/situations that perfectly characterize a cooking environment have been classified: pots with lids, pots without lids, pots without lids and water boiling, pans without oil, pans with oil, kitchenware, users, and others (other types of objects that can be found in the kitchen, such as cleaning rags, mobile phones, etc.). Some examples of the objects to detect can be seen in Figure 3. These objects will be enough to implement an algorithm that can act on the cooktop depending on what is detected.

**Figure 4:**
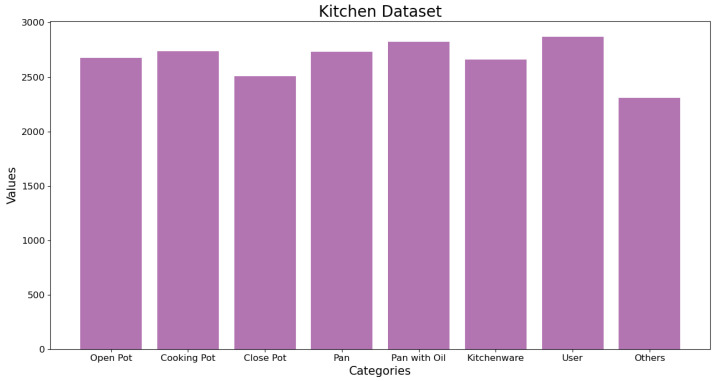
Histogram of the dataset.

Furthermore, images have been collected with different light environments simulating different times of the day and different seasons, trying to obtain the most universal dataset possible. The model in which the experiments were carried out is in an industrial laboratory, which lacks natural light. However, it has several light sources. For this reason, when collecting the images, the lighting of the workplace was modified (more and less light) to make the images and, consequently, the algorithm more universal. In addition, as the creators of YOLOv5 recommend, all the classes have been balanced (see Figure 4) and background images have been added (approximately 9%) to the dataset, which are images without objects that are used to reduce the number of false positives to improve the results.

The dataset has been divided into two folders: training (90%) and validation (10%), as shown in Table 1. After training and validation, real-time tests are performed to evaluate the algorithm data and determine if it is sufficient for the real application or, on the other hand, it is necessary to modify the dataset and re-train the model.

### 3.3. Data Collection and Labelling

**Figure 5:**
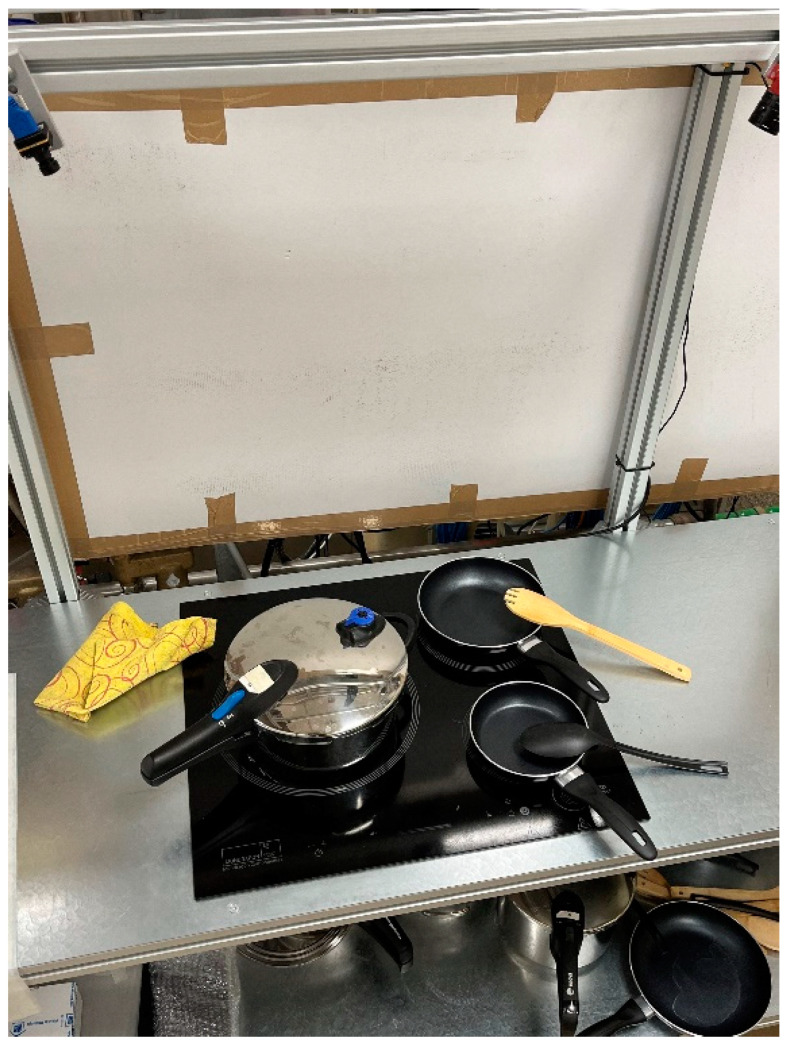
Functional mock-up where the experiments have been carried out.

On the one hand, the images used to train the object detection algorithm have been obtained using an Allied Vision camera placed in a functional model of a real kitchen. The cooking environment can be seen in Figure 5. A Python program has been used to capture the images with the help of Allied Vision’s Vimba Python API [32].

**Figure 6:**
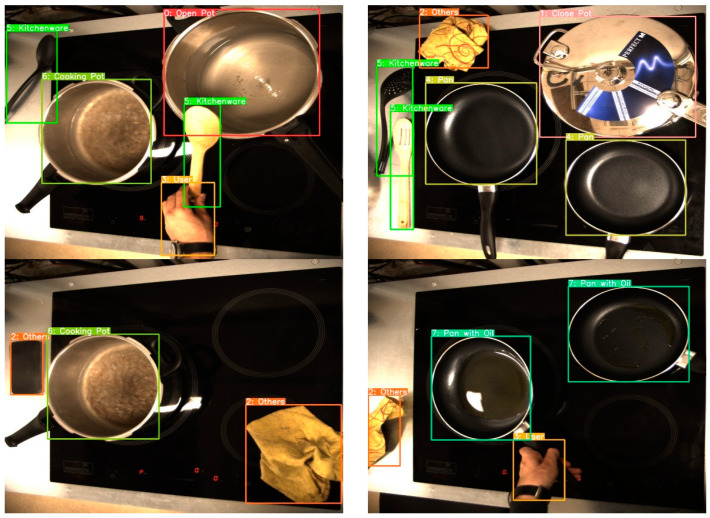
Examples of labelled images.

On the other hand, data annotation (also known as labelling) is a fundamental component of DL, because the accuracy of the AI models is directly correlated to the quality of the data used to train them [3,33]. Data labelling refers to the process of adding tags or labels to raw data. The images from the dataset mentioned above were manually labelled using the YOLO-Label annotation tool [34]. YOLO-Label is an easy and simple tool for labelling object bounding boxes in images using the YOLO label format, which consists of five columns for each object (category or object class, x, y, width, and height) in a TXT file. The last four columns must be normalized between zero and one so they can be correctly used for training. Some of the labelled images can be seen in Figure 6.

### 3.4. Data Augmentation

Data augmentation is the artificial generation of data by means of transformations of the original data. This allows for increasing the size and diversity of our training dataset. In general, having a large dataset is crucial for the performance of ML and DL models.

Different techniques can be applied to a dataset composed of images to increase the number of data:

- Geometric transformations
- Color space transformations
- Image noise
- Image mixing
- Random erasure

**Figure 7:**
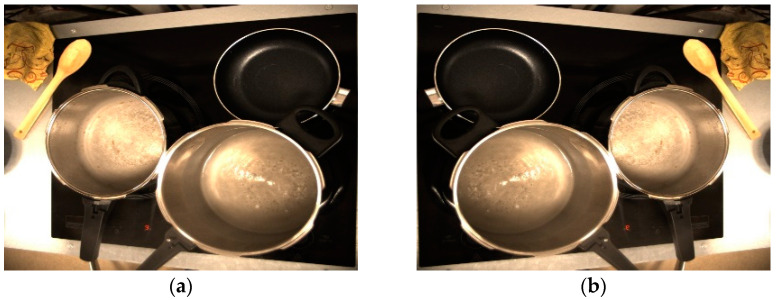
Data augmentation technique 1. Position manipulation: image flipping. (**a**) Original; and (**b**) Horizontal flip.

In this case, two of the mentioned techniques will be used, which, although they are two of the simplest, make a lot of sense in a cooking environment. Firstly, a horizontal flip of the images is used, which is one of the position manipulation techniques. The horizontal flip technique returns an image flipped along the y-axis. This makes a lot of sense because in some kitchens the camera may have to be placed on the opposite side to the one used in this work. An example of this technique can be seen in Figure 7.

**Figure 8:**
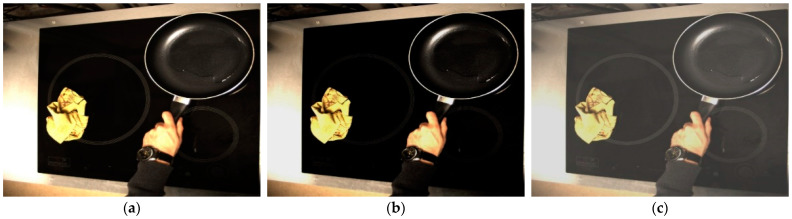
Data augmentation technique 2. Color jitter: random brightness and contrast. (**a**) Original; (**b**) Random brightness; and (**c**) Random contrast.

Secondly, the values of brightness and contrast will be changed randomly, altering the color properties of an image by changing its pixel values. On the one hand, brightness will make an image darker or lighter compared to the original. To change the brightness of the image, it is first converted to Hue, Saturation and Value (HSV) and then the V parameter is changed randomly up to ±20%. On the other hand, contrast is defined as the difference between the maximum and minimum pixel intensity in an image. Therefore, to modify the contrast in an image, the distance between the maximum and minimum pixel intensities must be modified. For each image, this distance is changed randomly. This technique will allow for universalization of the developed algorithm, as the illumination of the cooking environment as well as the images collected by different cameras can be different from the point of view of illumination. The new image will, therefore, be different from the point of view of luminance and color. Figure 8 shows this second data augmentation technique.

The idea is to generate different datasets using these image augmentation techniques and compare the results obtained with each of the sets to identify if it is possible to improve the training results obtained with the clean image dataset.

## 4. Results

In this section, the results of the proposed algorithm, which aims to modify and improve user experiences in the cooking environment, will be discussed. On the one hand, the results of the object detection algorithm will be studied, comparing the results obtained for the different data augmentation techniques mentioned in Section 3 and different object detection models. On the other hand, the results of the identification of interesting situations based on the object detection algorithm will be detailed and illustrated.

### 4.1. Object Detection Results

For the first experiment of the proposed system, the second smallest and fastest YOLOv5 model was chosen (YOLOv5s). With this YOLOv5 model, the different data augmentation techniques will be compared. The official YOLOv5 guide is used for the training of custom data, following all the steps for training and validating the performance of the model [35]. For the next experiment of the system, different YOLOv5, YOLOv6 and YOLOv7 models were used. The comparison considers the accuracy of the model as well as its runtime. For the visualization of the training results, Tensorboard is used, which is the TensorFlow visualization toolkit. The device used in this experiment is a laptop with an Intel Core i7-11800H CPU and a NVIDIA GeForce RTX 3070 GPU. The software environment is CUDA 11.6 and Python 3.8. The parameters of the training network are shown in Table 2. The learning rate and momentum are gradient descent optimization algorithm parameters. The learning rate controls how much the weights of the model are adjusted with respect to the loss function. The momentum allows for accumulating the gradient of previous steps to determine the direction to follow in the stochastic gradient algorithm (SGD) to optimize the model weights. The batch size is the number of samples the algorithm takes to train the network in each iteration, because when working with large amounts of data, you cannot pass all the data at once to the algorithm. Finally, the number of epochs is the number of times the entire dataset is passed through the network.

To evaluate the performance of the models with the developed dataset, the precision (*P*), recall (*R*), and average precision (*AP*) were calculated and compared. The metrics were calculated with the help of the following equations. The precision is the ratio of the number of true positives to the total number of positive predictions. The recall measures how well the model can find true positives out of all the predictions. The *AP* defines the proportion of the correct detections to the sum of the correct detections and false detections of objects, and the *mAP* is the average identification accuracy of all the classes.

(1) $$P=\frac{TP}{TP+FP}$$

(2) $$R=\frac{TP}{TP+FN}$$

(3) $$AP=\overset{N}{\underset{k=1}{\sum }}Precision\left(k\right)\;\Delta Recall\left(k\right)$$

(4) $$mAP=\frac{1}{N}\overset{N}{\underset{i=1}{\sum }}AP_{i}$$

where *TP*, *FP* and *FN* refer to the true positives, false positives, and false negatives, respectively. True positives are positive samples with correct classification, false positives are negative samples with incorrect classification, and false negatives are positive samples with incorrect classification. The results for all the classes for the first dataset are shown in Table 3.

Furthermore, in Table 4, the results of the different data augmentation techniques are shown. Dataset 1 is the smallest dataset and is composed of clean images (images that have not been processed with data augmentation techniques). In dataset 2, original images with horizontally flipped images are used. In dataset 3, original images with images in which the brightness and contrast were randomly changed have been used for training. Finally, dataset 4 contains a mix of images from the different data augmentation techniques, up to 15,000 in total.

**Figure 9:**
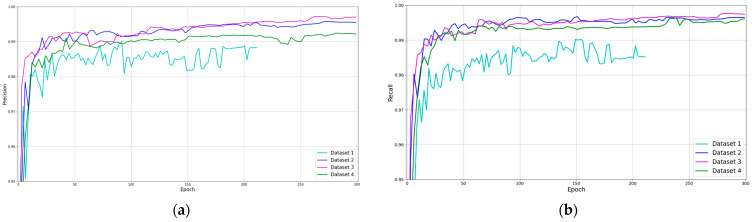

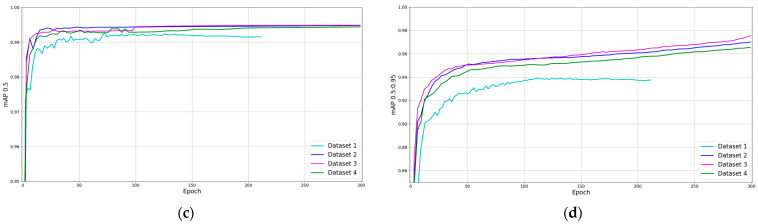
Data augmentation technique results comparison (precision, recall and *mAP*). (**a**) Precision; (**b**) Recall; (**c**) *mAP* 0.5; and (**d**) *mAP* 0.5:0.95.

Moreover, Figure 9 shows the training process for the three used datasets. The precision, recall and *mAP* metrics have been compared. As can be seen, the training of the first dataset stops when it takes a little more than 200 epochs, which means that the model has not learned for more than 100 epochs. Furthermore, the models trained with the other three datasets could keep learning if the training would continue. A simple analysis of the results shows that applying data augmentation techniques gives more than a 1% increase in the accuracy of the model. In addition, the three training sessions with data augmentation offer similar results.

Furthermore, different models were compared to see their generalization ability to detect common kitchen objects (Table 5). The dataset used for the training and validation of the models was the dataset composed of mixed images from the different data augmentation techniques (dataset 4).

To compare the different models, the epochs have been reduced to 150 to reduce the training time. Moreover, the batch-size training parameter of some models has been reduced to 16 or 8, so the training is done with the computer GPU. On the one hand, the results show that although all the models offer similar results, the YOLOv7, YOLOv6s and YOLOv6m models have better precision and *mAP*. However, the results do not vary considerably between the different architectures: the maximum difference between all the models is less than 1%. On the other hand, another interesting parameter is the speed of the algorithms, which is important too in real-time applications. The calculation of the FPS has been performed with the evaluation codes associated with each of the models. The FPS refers to the inverse of the time an algorithm takes to make a prediction (the sum of the pre-processing, inference time and non-max suppression). This time depends on the number of parameters, the architecture of the model and the image size. It has, therefore, been decided to strike a balance between accuracy and FPS. Due to its good results, it has been decided to use the YOLOv5s model for the application, although any of the models could be used.

### 4.2. Object Detection Post Algorithm

As mentioned in Section 1, the aim of this work is to try modifying and improving the user experience in the cooking environment. Therefore, the idea is to detect different situations and act consequently: the action will consist of manipulating the cooking heaters or displaying certain messages on the screen. However, messages to a connected device such as a mobile phone or a smartwatch, as well as different sound alarms, could be enabled.

Everything starts with the execution of the object detection algorithm. For each input image, the results are the labels of the detected objects as well as their positions in the image. The idea is to use this information to identify some relevant situations.

**Figure 10:**
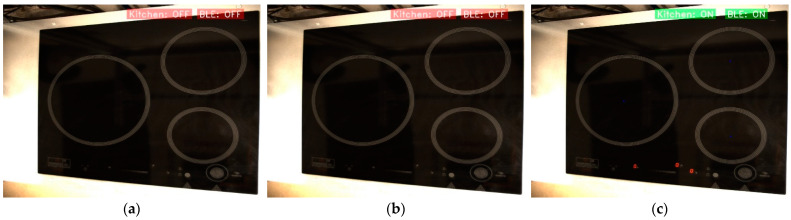
Kitchen BLE connection sequence. (**a**) Kitchen and BLE off; (**b**) Kitchen on and connecting BLE; and (**c**) Kitchen and BLE on.

Firstly, to be able to act on the cooker, it will be necessary to enable connectivity with an external device. This is done via a BLE interconnection. As shown in the flowchart in Figure 2, before starting the object detection, the program can detect and connect to cooktops with this type of technology. Figure 10 shows what a connection sequence looks like. On the one hand, in Figure 10a, the cooktop is not yet switched on, so it is impossible to make the BLE connection. On the other hand, in Figure 10b, the cooker is switched on, which means that it is possible to make the connection, and the computer starts searching for compatible devices. Finally, in Figure 10c, the cooker is on and the connection has been established.

**Figure 11:**
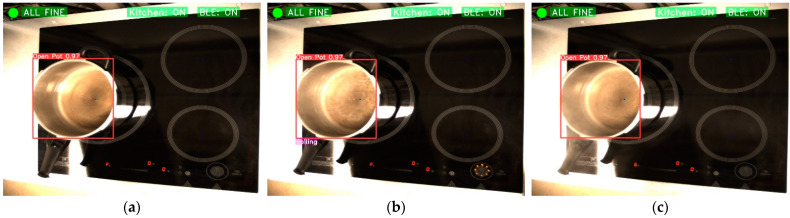
Boiling situation identification. (**a**) Open pot with maximum power but without boiling; (**b**) Open pot with boiling; and (**c**) Open pot without boiling and power has been reduced.

After the connection of the computer to the cooktop, the detection of objects and the consequent identification of situations of interest begins. Firstly, when the pot has no cover, the algorithm will be able to determine if there is water boiling in it. If cooking water is detected, a command will be sent to the cooker to reduce the power of the corresponding heater. Figure 11 shows the boiling water identification sequence. In Figure 11a, water is cooked on one of the rings. When it is detected that the water is already boiling, a command is sent to the cooker to reduce the power, as in Figure 11b. Once this command is accepted, the power of the heater is reduced, as shown in Figure 11c.

**Figure 12:**
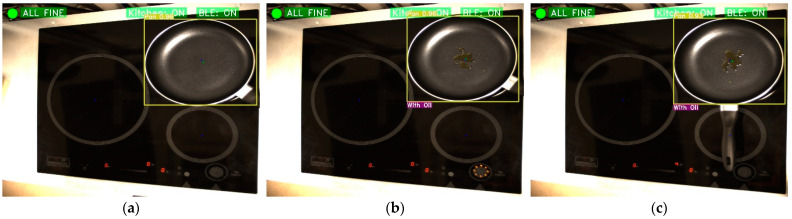
Pan with oil identification. (**a**) Normal pan without oil; (**b**) Identification of pan with oil; and (**c**) Pan with oil and power has been increased.

On the other hand, frying pans that do have oil in them will also be identified so that the burner can be switched on automatically. The operation sequence can be seen in Figure 12. Figure 12a shows a frying pan on top of a ring. Figure 12b identifies that there is oil in this frying pan and sends a command to increase the fire. Finally, Figure 12c shows that once the command has been accepted, the fire power has been increased.

As can be seen from these two examples, after detecting these situations, it will be possible to act in the kitchen by automatically switching on, manipulating, or switching off heaters. However, these are two specific situations, but the idea could be extrapolated to other events, such as the detection of food in different states of cooking, or even smoke or fire.

Another of the tasks performed by the developed algorithm is the recommendation of the best heaters depending on the cooking object (in this case, pans or pots). By calculating the size of the heater, which is related to the distance to the camera, and the cookware used for cooking, the algorithm can recommend the best position for it. This recommendation will also depend on the objects already available in the cooking environment. For this purpose, a matrix with scores has been generated that relates each of the heaters to the object and its size. In addition, the system can tell the user if the object is correctly arranged on the ring to make full use of all the cooking power. One of the advantages of this task is that energy efficiency can be controlled and optimized, and users can be supported in their daily cooking.

**Figure 13:**
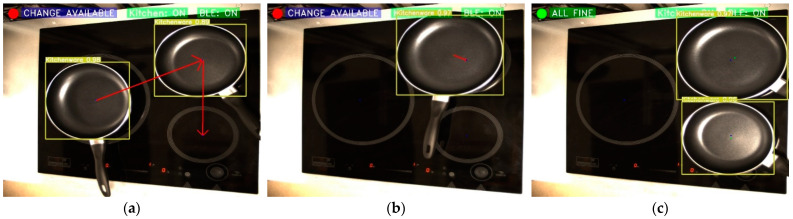
Example of a recommendation for the best kitchen heater. (**a**) Both pans can be repositioned; (**b**) The pan is in the right heater, but its position can be improved; and (**c**) The pans are perfectly placed.

In Figure 13, it can be seen how the burner recommendation algorithm works. In Figure 13a, for example, all the objects could be better placed, so the user is recommended to move them to the best spots. In Figure 13b, it is noted that the object is in the correct heater but not properly centered. Finally, in Figure 13c, the frying pans are correctly placed.

**Figure 14:**
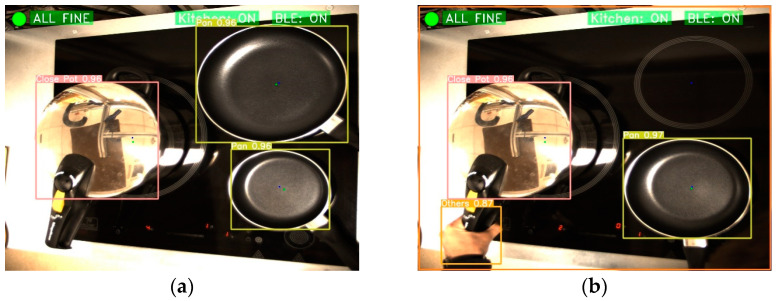
User cooking identification. (**a**) Not user; and (**b**) User manipulating.

In addition, the system can detect when there is somebody in the cooking environment. This makes it possible, for example, to switch off the cooker if it has not detected a user for a long time so that risky situations can be avoided (see Figure 14).

**Figure 15:**
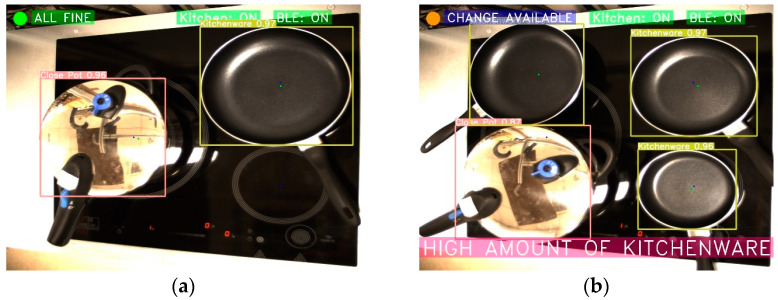
Example of high amount of kitchenware. (**a**) Good situation; and (**b**) Identification of high amount of kitchenware.

On the other hand, if the system detects a high amount of kitchenware or kitchen objects in the cooking environment, a message is sent so the user can remove them from the cooking environment. This is especially useful for groups with reduced visibility. Figure 15 shows how this is detected.

**Figure 16:**
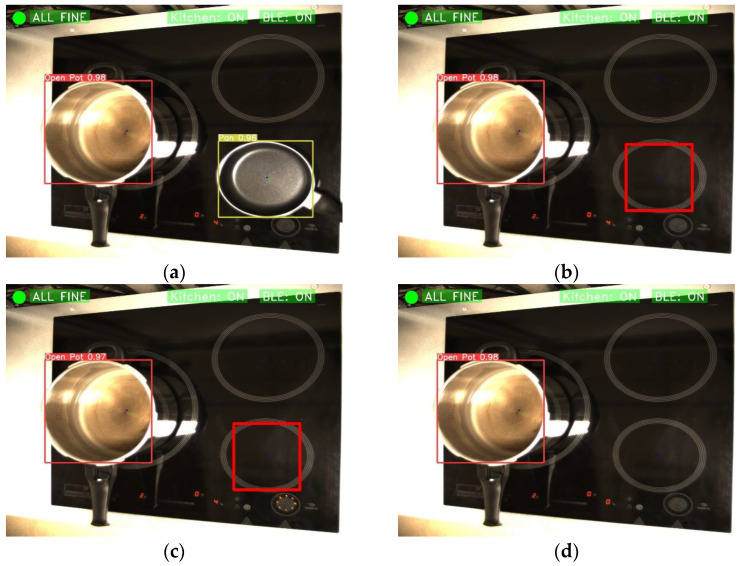
Example of high amount of kitchenware. (**a**) Start situation of the example; (**b**) The algorithm has detected that a burner is on with nothing on it; (**c**) The command to switch off the heater is sent; and (**d**) The burner is off.

Moreover, the algorithm can detect when a heater is on without a pan or pot on it. This is useful, for example, in situations where a heater is left on accidentally or forgotten. Perhaps we are used to the fact that induction cookers automatically turn off the fire when there is nothing on it. However, this is not the case for all types of cookers: radiant cookers continue to supply power even when nothing is on them. In Figure 16, an example of how the automatic detection and shutdown works is shown. In this case, the shutdown happens instantly, although timers could be configured to cover different situations.

**Figure 17:**
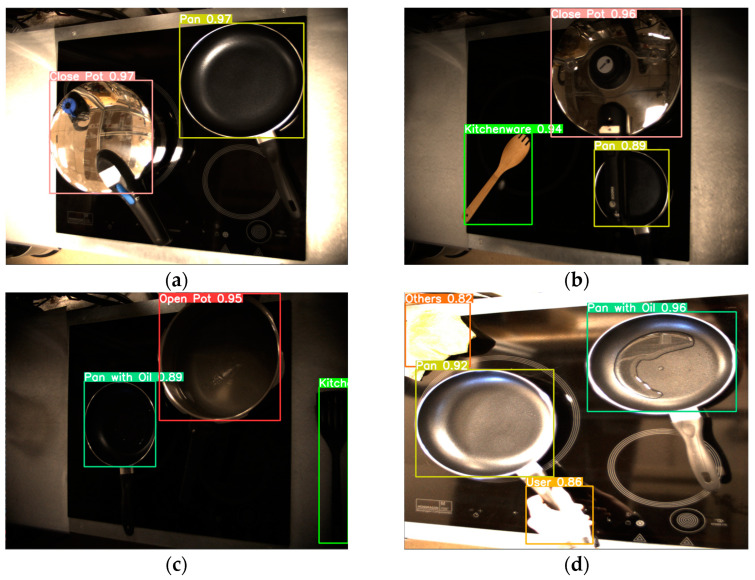
Developed algorithm under different light conditions. (**a**) Wider framing and lighter; (**b**) Wider framing and less light; (**c**) Wider framing and low light; and (**d**) More light and tighter framing.

Finally, the algorithm has been tested under different light conditions and with different image crops (see Figure 17). The idea is to test how the algorithm works with images that are different from the ones used for training. The algorithm still works correctly even though the framing of the cooktop is different from the original and the lighting differs from the normal one.

## 5. Discussion

The performance of the different YOLO algorithms was briefly discussed in Section 4.1. All the algorithms give results above 98% in accuracy, which is sufficient for the developed application. Another analyzed parameter was the inference speed of the algorithm, which resulted in more than 50 FPS for all the models. However, in the developed program, this is limited by the frame rate of the camera used for this project, which is limited to a maximum of 5 FPS. As the image capture and posterior analysis have been carried out in parallel, the execution time has not been a problem. On the other hand, the final idea is to implement such algorithms on low-cost hardware, so preference has been given to lighter models whose computational time is shorter.

In addition, some examples of situations that have been considered interesting for users have been shown in Section 4.2. These situations include various scenarios, such as identifying hazardous conditions, improving energy efficiency, and assisting in the cooking environment. Although these situations are simple, they show how the developed method works and could be extrapolated to other situations, such as fire detection, identification of foreign objects on burning fires or the complete automation of a recipe.

One of the problems we have encountered when communicating with the cooktop and the computer is that if multiple commands are sent for different heaters at the same time, only the last command would be accepted and all the previous information would be lost. As it is impossible to give two commands at the same time, e.g., turn the large ring up to eight and turn off the small one, a queuing system has been generated, in which the instructions wait until the user accepts each individually.

As mentioned, there are a few examples in the literature of the use of deep learning algorithms for application in the cooking environment. In addition, to the best of our knowledge, this is the first time that research has employed DL to automatically control the cooktop. For this reason, we intended to contribute and open the theme for future research and application of DL algorithms not only in the cooking environment, but also in different household appliances.

To conclude this section, the contributions of this paper will be compared with others where similar themes are addressed. In [2], a dataset for kitchen object segmentation is generated. In addition, a method for generating synthetic images is proposed. However, many images are unusable and differ considerably from a realistic environment. In total, they have a dataset of 719 images, half of which are real and half of which are synthetic. In [36], an RGB-D dataset of nine kitchen scenes is presented. The dataset contains more than 6000 images in which multiple objects in different environments of a kitchen are labelled. The images are taken by a person in movement and are labelled with bounding boxes and in the 3D point cloud. In [37,38], the large-scale EPIC-KITCHENS dataset is described. It has more than 20 million images with different types of annotations (for example, activity classification, object detection or image segmentation) on 45 kitchens, and the images have been captured with a head-mounted camera. However, not all the objects identified in this work are covered, so it is not useful for this application. On the other hand, our dataset has more than 7500 clean images, more than in some of the examples seen, and they have been obtained in a static way, which can be very useful for other types of kitchens with similar characteristics. On the other hand, our dataset can even be combined with other datasets to improve the results and cover cases not considered in this work.

Finally, in [39,40,41], cooking activities using ML and DL techniques are classified. These models are useful for recognizing situations such as cutting, baking, washing, or cooking. However, these examples lack feedback on the cooktop as is done in our work. Even in [42], a method is proposed to build a real-time detection algorithm for the prevention of cooktop fires, but they do not act on the cooker either.

## 6. Conclusions

The aim of the present work was to develop an algorithm that, based on a deep learning object detection model, could help users in the cooking environment. A real-time and accurate object detection method based on the YOLOv5 model was proposed to modify and improve users’ cooking experience. The developed algorithm can identify multiple interesting situations. Some of these are the detection of boiling water or oil in pans and pots, recommendations, correct positioning of cookware on the heaters, and burners with nothing on them. Based on these situations, the cooker can automatically be controlled by manipulating its heaters’ power, or users can be alerted by sound alarms or messages on their mobile phones.

To train the model, different datasets were created using different data augmentation techniques. The use of these techniques demonstrates that the model has improved its results. Different versions of YOLO algorithms were also tested, and it was determined that YOLOv5s is good and fast enough to be applied in the proposed system. YOLOv5s was demonstrated to be capable of identifying common objects in a cooking environment with a precision and recall of over 98% in the test sets.

Furthermore, after comparing the different models, we have seen that all of them offer similar results for the application we have developed. In this way, we have realized that with lighter models and shorter training sessions, very accurate algorithms can also be obtained. In addition, it has been considered that the generated dataset has an excessive amount of training data, and that with a smaller initial dataset, the results can be nearly replicated.

In summary, the YOLOv5 model detection network combined with multiple data augmentations can accurately and quickly detect common cooking environment objects, recognize interesting situations and automatically interact with the cooktop.

Future work includes the implementation of the model in integrated electronics, such as a microcontroller, with the main objective of developing a cost-effective solution to keep the algorithm running in real time. In addition, new categories of objects will continue to be added to the dataset to satisfy the new identified needs. Finally, this could help users to make pre-defined recipes interactive [43], or even bring us closer to automating the entire cooking process.

## Figures and Tables

**Table 1:** Dataset training and validation distribution.

Set	Number of Images
Train	6982
Validation	728
Test	Real-time validation on functional mock-up
Total	7710

**Table 2:** Parameters of training.

Parameters	Value
Image size	640 × 640
Learning rate	0.01
Momentum	0.937
Batch size	32
Total epoch	300

**Table 3:** Results of the clean dataset.

Class	Images	Instances	Precision	Recall	*mAP* 0.5
All	727	1920	0.984	0.985	0.992
Open Pot	727	245	0.988	0.988	0.995
Close Pot	727	239	0.994	1	0.995
Cooking Pot	727	235	0.982	0.996	0.994
Pan	727	258	0.981	1	0.993
Pan with Oil	727	275	0.998	1	0.995
Kitchenware	727	218	0.976	0.954	0.981
User	727	272	0.963	0.974	0.99
Others	727	238	0.991	0.971	0.993

**Table 4:** Results for the different data augmentation datasets.

Dataset	Class	Images	Precision	Recall	*mAP* 0.5
Dataset 1	All	727	0.984	0.985	0.992
Dataset 2	All	1463	0.996	0.996	0.995
Dataset 3	All	2187	0.997	0.997	0.995
Dataset 4	All	1424	0.992	0.996	0.994

**Table 5:** Results for different object detection models.

Model	Params (M)	Batch Size	Precision	Recall	*mAP* 0.5	FPS	Inference Time (ms)	Training Time (h)
YOLOv5n	1.9	32	0.988	0.992	0.994	84	9.3	5.48
YOLOv5s	7.2	32	0.991	0.993	0.994	80	10.3	5.88
YOLOv5m	21.2	16	0.992	0.995	0.994	73	11.9	10.45
YOLOv5l	46.5	8	0.993	0.996	0.994	61	14.7	17.48
YOLOv6n	4.3	32	0.984	0.990	0.994	43	17.2	6.07
YOLOv6s	17.2	16	0.994	0.990	0.997	42	20.6	9.38
YOLOv6m	34.3	8	0.995	0.990	0.997	39	23.5	20.71
YOLOv7	36.9	16	0.992	0.992	0.997	61	15	15.15

## Data Availability

The data presented in this study are available on request from the corresponding author.
